# Supplementary figures and images for: Intra and inter-rater reproducibility of the Remote Static Posture Assessment (ARPE) protocol’s Postural Checklist
Source: PLoS One. 2024 Feb 9;19(2):e0297506. doi: 10.1371/journal.pone.0297506 (PMC10857740; doi:10.1371/journal.pone.0297506)

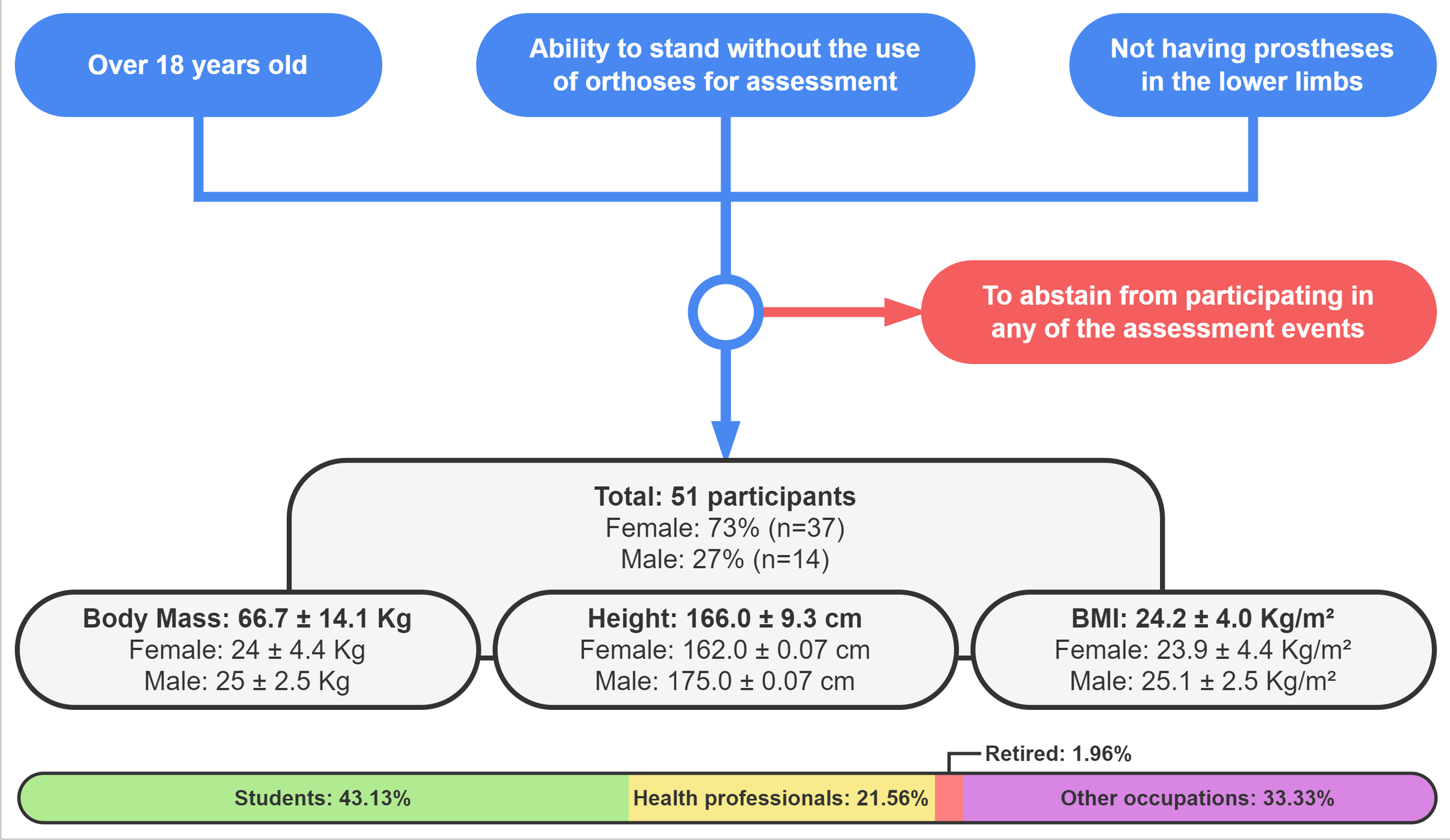

Supplement: S1 Appendix — (TIF) [file pone.0297506.s001.tif]

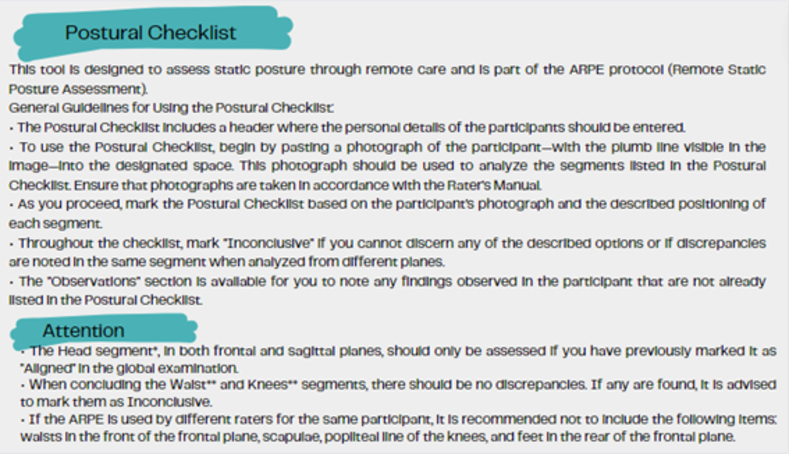

Supplement: S2 Appendix — (TIF) [file pone.0297506.s002.tif]
